# Hypoxia‐inducible factor (HIF)‐1α‐induced regulation of lung injury in pulmonary aspiration is mediated through NF‐kB

**DOI:** 10.1096/fba.2021-00132

**Published:** 2022-01-12

**Authors:** Madathilparambil V. Suresh, George Yalamanchili, Tejeshwar C. Rao, Sinan Aktay, Alex Kralovich, Yatrik M. Shah, Krishnan Raghavendran

**Affiliations:** ^1^ Department of Surgery University of Michigan Ann Arbor Michigan USA; ^2^ Department of Cell, Developmental, and Integrative Biology The University of Alabama at Birmingham Birmingham AL, USA; ^3^ Molecular & Integrative Physiology University of Michigan Ann Arbor Michigan USA

**Keywords:** CASP, HIF‐1α, IVIS, NF‐kB, Type II AEC

## Abstract

Aspiration‐induced lung injury is a common grievance encountered in the intensive care unit (ICU). It is a significant risk factor for improving ventilator‐associated pneumonia (VAP) and acute respiratory distress syndrome (ARDS). Hypoxia‐inducible factor (HIF)‐1α is one of the primary transcription factors responsible for regulating the cellular response to changes in oxygen tension. Here, we sought to determine the role of HIF‐1α and specifically the role of type 2 alveolar epithelial cells in generating the acute inflammatory response following acid and particles (CASP) aspiration. Previous studies show HIF‐1 α is involved in regulating the hypoxia‐stimulated expression of MCP‐1 in mice and humans. The CASP was induced in C57BL/6, ODD‐Luc, HIF‐1α (+/+) control, and HIF‐1α conditional knockout (HIF‐1α (−/−) mice). Following an injury in ODD mice, explanted organs were subjected to IVIS imaging to measure the degree of hypoxia. HIF‐1α expression, BAL albumin, cytokines, and histology were measured following CASP. In C57BL/6 mice, the level of HIF‐1α was increased at 1 h after CASP. There were significantly increased levels of albumin and cytokines in C57BL/6 and ODD‐Luc mice lungs following CASP. HIF‐1α (+/+) mice given CASP demonstrated a synergistic increase in albumin leakage, increased pro‐inflammatory cytokines, and worse injury. MCP‐1 antibody neutralized HIF‐1α (+/+) mice showed reduced granuloma formation. The NF‐κB expression was increased substantially in the HIF‐1α (+/+) mice following CASP compared to HIF‐1α (−/−) mice. Our data collectively identify that HIF‐1α upregulation of the acute inflammatory response depends on NF‐κB following CASP.

**Abbreviations**
A549: human lung epithelial cells
ACID: hydrochloric acid
AEC: alveolar epithelial cell
ALI: acute lung injury
AM: alveolar macrophage
ARDS: acute respiratory distress syndrome
BAL: bronchoalveolar lavage fluid
BSA: bovine serum albumin
CASP: combination of acid and small gastric particles
CCR2: C‐C Motif Chemokine Receptor 2
CCR2: chemokine receptor 2
ELISA: enzyme‐linked immunoassay
HGF: hepatocyte growth factor
HIF‐1α: hypoxia‐inducible factor 1 alpha
HO‐1: heme oxygenase 1
HRE: hypoxia‐response elements
ICU: intensive care unit
IL‐1β: interleukin beta
IL‐6: interleukin 6
KC: keratinocyte chemoattractant
LC: lung contusion
MIP‐2: macrophage inflammatory protein
NF‐κB: nuclear factor kappa‐light‐chain‐enhancer of activated B cells
ODD‐Luc: oxygen‐dependent domain of HIF‐1α linked with luciferase
PBS: phosphate‐buffered saline
P–V: lung pressure–volume curve
SNAP: small non‐acidified food particles
TLC: total lung capacity
TNF‐α: tumor necrosis factor‐alpha
VAP: ventilator‐associated pneumonia
WES: capillary western immunoassay

## 1 INTRODUCTION

The aspiration of gastric contents is a significant risk factor for acute lung injury and acute respiratory distress syndrome (ALI/ARDS).^1^, ^2^, ^3^ Pulmonary aspiration includes inhalation of food, stomach acid, or saliva into the lungs.^4^, ^5^, ^6^ The aspirate content is highly variable and may include secretions, blood, bacteria, liquids, and food particles. Inhalation of any particles mentioned above could progress to ALI or ARDS.^2^, ^7^, ^8^, ^9^, ^10^ Gastric aspiration is common in patients deemed “unconscious” or “unresponsive” with a reported occurrence of one in every 2000–4000 anesthetized cases.^11^ Acute pulmonary injury in rodents has previously been shown to lead to inflammation‐mediated responses that differ in magnitude and pattern depending on the type of aspiration, such as hydrochloric acid (ACID), small non‐acidified food particles (SNAP), or a combination of acid and small gastric particles (CASP).^11^, ^12^, ^13^, ^14^ The severity of lung injury following CASP ranges from mild, subclinical pneumonitis to progressive respiratory failure with significant morbidity and mortality.^5^, ^12^, ^13^

ARDS is characterized by widespread lung injury and inflammation and is clinically seen as shortness of breath with hypoxemia. Hypoxia is a hallmark of lung injury and is commonly seen in ARDS cases.^14^, ^15^, ^16^ HIF‐1α is a dimeric protein complex that plays an integral role in the body's response to low oxygen concentrations. It is a master regulator of hypoxic/ischemic vascular responses, driving transcriptional activation of hundreds of genes involved in vascular reactivity, angiogenesis, arteriogenesis, erythropoiesis, energy metabolism, and cell survival.^15^, ^17^, ^18^, ^19^, ^20^ Under hypoxic conditions, HIF‐1α is stabilized and subsequently translocated to the nucleus, where it dimerizes with HIF‐1β and activates downstream target genes containing hypoxia‐response elements (HRE) within their promoter or enhancer regions.^21^, ^22^, ^23^, ^24^, ^25^ There are many factors, such as inflammation and hypoxia that can stimulate and activate HIF‐1α‐related pathways. A recent study shows that HIF‐1α/PDK1‐mediated glycolytic reprogramming is a critical metabolic alteration that stimulates myofibroblast differentiation and fibrotic progression.^26^ Our laboratory has demonstrated that type II alveolar epithelial cells (AEC), instead of innocent bystanders in lung injury, primarily direct the inflammatory response to lung contusion (LC). Additionally, we reported that hypoxic activation of HIF‐1α in AEC is critical to initiating the inflammatory response after LC.^27^, ^28^

Several reports indicate the protective nature of MCP‐1/CCL2 during an acute pulmonary injury in LC.^29^ We have shown that elevated levels of MCP‐1 in the bronchoalveolar lavage (BAL) are associated with improved oxygenation in LC injury in rats.^30^ Hideaki et al. reported that the administration of MCP‐1/CCL2 increased the number of alveolar macrophages that ingest neutrophils and hepatocyte growth factor (HGF) levels in BAL fluid and eventually attenuated lung tissue injury. They also reported immunoreactive MCP‐1 proteins are detected in alveolar type II epithelial cells and alveolar macrophages (AMs) only after *P. aeruginosa* infection.^31^ Recently, we showed that in the absence of the C‐C Motif Chemokine Receptor 2 (CCR2), the extent of mechanical damage in ALI after LC is worsened and prolonged. The same response was observed in rats injected with anti–CCL2 antibody.^29^ We have previously reported increased mortality, persistent inflammation, and decreased granulomatous compartmentalization in MCP‐1/CCL‐knockout mice injured with CASP.^32^ However, in this specific study (HIF‐1α (+/+) and HIF‐1a (−/−) mice), we did not notice any mortality at any time point following CASP.

Nuclear factor‐κB (NF‐κB) is a critical transcription factor required to express various pro‐inflammatory molecules that play a crucial role in the pathogenesis of acute lung inflammation.^33^ In previous studies, the activation of NF‐κB has been linked to acute lung injury. It is a proximal step in initiating neutrophilic inflammation in animal models.^33^, ^34^, ^35^ However, the precise regulation of NF‐κB activation on HIF‐1α in type 2 AEC in the injured lung is unknown. We examined NF‐κBp65 activation in HIF‐1α (+/+) and HIF‐1α (–/–) mice subjected to lung contusion. Our results suggest that HIF‐1α downregulation of the acute inflammatory response is in part dependent on NF‐κBp65.^14^

For this study, a combination of acid and small non‐acidified gastric food particles simulated gastric aspirates observed in a clinical setting. In this manuscript, we set out to determine the role of HIF‐1α in combined acid plus minor particles‐induced lung injury and examine the effects of HIF‐1α‐induced human lung epithelial cell injury and cell death. We used HIF‐1α triple transgenic conditional knockout mice specific in type II AEC to confirm and characterize hypoxic AEC's fate in the pathogenesis of acid plus food particle‐induced lung injury.

## 2 MATERIALS AND METHODS

### 2.1 Animals

Here, we used male and female age‐matched (7–8 weeks) wild type (C57BL/6), HIF‐1α triple transgenic conditional knockout mice specific for type II alveolar epithelial cells (AEC), SP‐C‐rtTA_/tg/(tetO)7‐CMV‐Cretg/tg/HIF‐1α _*flox*/*flox* mice, and hypoxia reporter mice with the oxygen‐dependent domain of HIF‐1α linked with luciferase (ODD‐Luc) (Jackson Laboratories). All procedures were approved by the Institutional Animal Care and Use Committee (IACUC) at the University of Michigan and complied with the state, federal, and National Institute of Health regulations.

### 2.2 In Vivo Imaging System (IVIS) of ODD‐Luc mice

ODD‐Luc transgenic mice, uninjured and CASP, were given intraperitoneal (i.p.,) injection of a mixture of luciferin (50 mg/kg) in sterile water. Twenty minutes later, mice were placed in a light‐tight chamber equipped with a charge‐coupled IVIS imaging camera (Xenogen). Upon injection of luciferin, the bioluminescent background of healthy tissues in the transgenic mice and bioluminescent signals from hypoxia were immediately measured non‐invasively with an IVIS spectrum imaging station that has been reported previously.^14^, ^27^, ^36^

### 2.3 HIF‐1α mice

Triple transgenic mice were created by mating HIF‐1α _*flox*/*flox* and SP‐C‐rtTA_/tg/(tetO)7‐CMV‐Cretg/tg transgenic mice. The mice, SP‐C‐rtTA_/tg/(tetO)7‐CMV‐Cretg/tg/HIF‐1α _*flox*/*flox*, are capable of respiratory epithelium‐specific conditional recombination in the floxed HIF‐1α gene upon exposure to doxycycline (a generous gift from John J. LaPress, Department of Biochemistry and Molecular Biology, Michigan State University). The HIF‐1α flox/flox mice were initially maintained in a C57BL/6 genetic background, whereas the SP‐C‐rtTA_/tg/(tetO)7‐CMVCretg/tg mice were generated in an FVB/N genetic environment.^14^, ^16^, ^27^, ^36^, ^37^

### 2.4 Doxycycline treatment to achieve recombination in HIF‐1α mice

Postnatal recombination was done by exposing lactating dams to feed containing doxycycline (625 mg/kg: Harlan Teklad) and drinking water (0.8 mg/ml, Sigma Chemical Company). Triple transgenic mice were then maintained on the same doxycycline‐containing food and water until 7 weeks of age. Doxycycline administration was terminated 7–10 days before gastric aspiration. These mice will be referred to as conditional knockout mice [HIF‐1α (−/−)]. Control animals used in the study were triple transgenic [SP‐C‐rtTA^−/tg^/(tetO)_7_‐CMV‐Cre^tg/tg^/HIF1α*^flox^* ^/^ *^flox^*] mice maintained on regular food and water ad libitum.^14^, ^16^, ^27^, ^37^

### 2.5 A murine model for gastric aspiration (CASP)

Gastric particles in all cases were prepared from the stomach contents of C57BL/6 WT mice, which were washed several times in normal saline, filtered through gauze, and sterilized by autoclaving.^5^ The acid component of gastric aspirates is frequently modeled by the intra‐tracheal installation of a combination of acid and small gastric particles (CASP) in animals (40 mg particles/ml, pH = 1.25). C57BL/6, ODD‐Luc, HIF‐1α (+/+), and HIF‐1α (−/−) mice were used for CASP administration. Mice were inoculated with the CASP suspension (30μl/mouse) or saline control via deep oral hypopharyngeal injection under isoflurane anesthesia.^4^, ^32^, ^38^

### 2.6 Administration of anesthetic, analgesic, and resuscitation

Animals were anesthetized by intraperitoneal injection of ketamine (80–120 mg/kg body weight) and xylazine (5–10 mg/kg body weight). Systemic analgesics were not used due to their effects on the immune system. In the event of severe respiratory distresses beyond 48 h, animals were humanely euthanized.^39^

### 2.7 Lung Pressure–Volume (P–V) curve

P–V was measured after blood samples were collected, and the mice were further exsanguinated by transection of the abdominal inferior vena cava. An 18‐gauge metallic cannula was inserted into the trachea through a midline cervical exposure. Animals were then connected to a SCIREQ Flexivent that allows for simultaneous animal ventilation and data capture as described previously.^25^, ^36^, ^40^

### 2.8 Albumin concentrations in bronchoalveolar lavage (BAL)

Albumin concentrations in the BAL samples were measured by ELISA using a polyclonal rabbit anti‐mouse albumin antibody and HRP‐labeled goat anti‐rabbit IgG (Bethyl Laboratories, Inc.) as described previously.^29^, ^41^

### 2.9 Determination of cytokine levels in BAL

Soluble concentrations of IL‐1β, IL‐6, TNFα, CCL2 (MCP‐1), CCL12 (MCP‐5), KC, and MIP‐2 in BAL were determined using ELISA. Mouse ELISA detection limit is as following: IL1‐beta: 15.6–1000 pg/ml; IL‐6: 15.6–1000 pg/Ml; TNFα: 31.2–2000 pg/ml; CCL2 (MCP‐1): 3.9–250 pg/Ml; CCL12 (MCP‐5): 31.2–2000 pg/ml; KC: 15.6–1000 pg/Ml and MIP‐2: 15.6–1000 pg/ml. Antibodies and recombinant cytokines for these assays were obtained from R&D Systems described previously.^29^, ^41^

### 2.10 Cell count (Cytospin)

BAL cells were centrifuged at 600× g for 5 min using a Cytospin II (Shandon Scientific), stained with Diff‐Quik (Dade Behring Inc.), and examined under a light microscope at 20× magnification as described previously.^29^

### 2.11 Histopathology

ODD‐Luc and HIF‐1α mice lung specimens were harvested at the time of death and subsequently fixed in 10% formalin, sectioned, and stained with hematoxylin and eosin as described previously.^29^, ^41^

### 2.12 Western blot

Mouse lungs were lysed in ice‐cold lysis buffer, mixed with a commercial sample buffer (Invitrogen), and heated at 95°C for 5 min. Samples were then electrophoresed on SDS–polyacrylamide gels, after which the gels were transferred to PVDF membranes. Blots were incubated overnight at 4°C with various primary antibodies. We used the HIF‐1α antibody (NB100‐134) from Novus Biologicals, LLC. The dilution is 1:500, and the molecular weight is 93 kDa. Primary antibody was followed by appropriate secondary antibodies. After washing, the signal was detected using a Super Signal chemiluminescent substrate Western Blotting reagent (Pierce Biotechnology) with the chemiluminescent‐sensitive film.^14^

### 2.13 Capillary western immunoassay (WES)

Western blot was performed on a WES system (004–600, Protein Simple) according to the manufacturer's instructions (SM‐W004, Protein Simple) and an anti‐rabbit detection module (DM‐001, Protein Simple). In brief, protein samples were 10‐fold diluted in sample buffer, mixed with Fluorescent Master Mix, and heated at 95°C for 5 min. The samples, blocking reagent, primary antibodies (1:100 dilution), HRP‐conjugated secondary antibodies, and chemiluminescent substrate were pipetted onto the separation module plate. The instrument default settings used were stacking and separation at 475 V for 30 min; blocking reagent for 5 min; primary and secondary antibody for 30 min; and luminol/peroxide chemiluminescence detection for 15 min. The resulting electropherograms were inspected to check whether automatic peak detection results required manual correction as described previously.^42^, ^43^, ^44^

### 2.14 Preparation and isolation of type 2 alveolar epithelial cells from mice

Crude lung cell suspensions were prepared from male HIF 1α (+/+) and HIF 1α (−/−) following CASP as previously described.^14^, ^27^, ^45^, ^46^ Briefly, mice were anesthetized and exsanguinated by opening the peritoneum and clipping the left renal artery, and the lungs were perfused with PBS. The lungs were filled with 1.5 ml dispase via the tracheal catheter and then allowed to collapse naturally. Then the lungs were immediately covered with crushed ice and incubated for 2 min. The lungs were then removed and placed in 2 ml dispase in a 12‐ml polypropylene culture tube, incubated for 45 min at room temperature, and put on ice until the next step. The lungs were transferred to DMEM with 0.01% DNase I in a 100‐mm Petri dish. The digested tissue was carefully collected from the airways with the curved edge of curved fine‐tipped forceps and gently swirled for 5–10 min. The suspension was successively filtered through 100 and 35 µm nylon filters and then 15 µm nylon mesh. The filtered cell suspension was centrifuged at 130× *g* for 8 min at 4°C and resuspended in the culture media. The cells were incubated with biotinylated anti‐CD‐32 and biotinylated anti‐CD‐45 antibodies for 30 min at 37^o^C.

Meanwhile, streptavidin‐coated magnetic particles were washed in a culture medium with a polypropylene culture tube using a magnetic tube separator. After incubation, the cells were centrifuged (130× *g* for 8 min at 4°C), resuspended in 7 ml DMEM, added to the magnetic particles, and incubated with gentle rocking for 30 min at room temperature. The tube was attached to the magnetic tube separator with adhesive tape for 15 min following the incubation. The cell suspension was aspirated from the bottom of the tube using a narrow‐stemmed transfer pipet, centrifuged, and resuspended in culture media. Cultures were incubated in a humidified, 10% CO_2_ chamber at 37°C and observed daily by phase‐contrast microscopy. The protocol utilizes floating cells (type II AEC), whereas immunologically active cells like macrophages and fibroblasts adhere to the culture dish. Next, cells were used to evaluate cell yield, viability, purity, and measure surfactant secretion.^14^, ^27^, ^45^, ^46^ The cultures were washed twice to collect the type II cells for RNA isolation.

### 2.15 Taq Man quantitative polymerase chain reaction

Total RNA was prepared from type 2 cells and whole lung lysate and reverse‐transcribed into cDNA using M‐MLV reverse transcriptase (Life Technologies Corporation). The cDNA was then amplified by real‐time quantitative TaqMan PCR using an ABI Prism 7700 sequence detection system. GAPDH was analyzed as an internal control. TaqMan gene expression reagents were mixed and used to detect all genes from applied biosystems (IL1‐β, IL‐6, TNF alpha, HIF‐1a, MIP‐2, NOS‐2, Arginase, FIZZ‐1, NF‐kB, and NLRP3) responsible for inflammation. Data were expressed as the fold‐change in transcript expression. The fold difference in mRNA expression between treatment groups was determined by software developed by Applied Biosystems (Applied Biosystems).^29^, ^41^

### 2.16 Immuno‐neutralization of MCP‐1 in HIF‐1α (+/+) and HIF‐1α (−/−) mice

The antibody injections (goat anti‐CCL2/JE/MCP‐1 (1:300, AF‐479‐NA) and control goat IgG (AB‐108) R&D Systems) were performed intraperitoneally, 48 h before CASP in sterile PBS (antibody concentration was 20 µg/mouse). Hiroshi et al. and others reported that this blocking antibody had been previously shown to efficiently neutralize the biological activity of MCP‐1 in vitro and in vivo.^47^, ^48^, ^49^

### 2.17 In‐vitro acid‐induced cell injury

A549 (Human lung epithelial) cells were plated at 4 × 10^5^ cells/well in 6‐well plates. After 24 h incubation, the media was changed to serum‐free DMEM followed by further culturing for 24 h. The cells were then incubated with HCl (pH 4.0) for 30 min at 37°C in 5% CO_2_. Control cells were exposed to PBS. After incubation with HCl, the medium was discarded; the cells were washed thrice with complete media to confirm the culture media's neutralization. Cells were then cultured with serum‐free media for an additional 4 h, and samples were collected.^33^, ^50^

### 2.18 Immunocytochemistry

A549 cells were seeded in 8‐chambered glass plates. After 24 h incubation, cells were treated with HCl (pH 4.0, DMEM) for 30 min at 37°C in 5% CO_2_. Control cells were exposed to phosphate‐buffered saline (PBS). After incubation with HCl, the acidified medium was discarded, and the cells were washed thrice with complete media to confirm neutralization. Cells were fixed by adding formaldehyde directly to the cells for 15 min at room temperature. Cells were washed with phosphate‐buffered saline (PBS), permeabilized with 0.2% Triton X‐100 (Sigma‐Aldrich), washed again with PBS twice, and blocked with PBS containing 1% bovine serum albumin (BSA). This cell was followed by incubation in HIF‐1α antibody (Santa Cruz Biotechnology) and NF‐kBp65 (Cell Signaling) followed by incubation in Alexa flour labeled secondary antibodies (Invitrogen) for 1 h. Nuclei were stained with DAPI. The cells on coverslips were then mounted in mounting media (Dako), and photomicrographs of the invasive sections were analyzed.^27^, ^51^

### 2.19 Statistical methods

Data are expressed as mean ± SEM. Statistical significance of data between two groups was analyzed using a two‐tailed, unpaired *t*‐test with Welch's correction. Data from more than two groups were analyzed using one‐way ANOVA with Turkey's multiple comparison tests (Graph Pad Prism 8.00).^52^ Data were analyzed at a significance level of *p* < 0.05.^14^, ^29^, ^41^

## 3 RESULTS

### 3.1 C57BL/6(WT) mice show upregulation of HIF‐1α expression, increased lung injury, and inflammation following CASP

**FIGURE 1:**
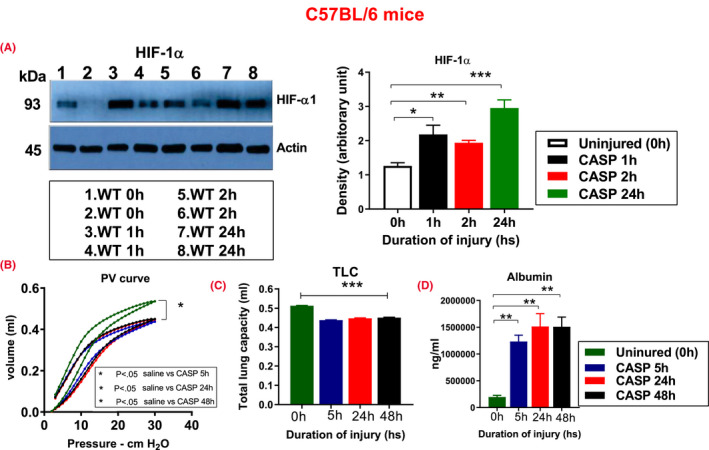
C57BL/6 showed increased HIF‐1α expression, lung injury following CASP. WT mice (*n* = 2) were subjected to CASP, and the lungs, along with uninjured saline control (0 h), were harvested at 0, 1, 2, and 24 h intervals. The HIF‐1α expression was measured (A). “Quasistatic” closed‐chest pressure‐volume behavior was measured at 5, 24, and 48 h after CASP administration (*n* = 6). CASP‐administered mice show increased injury compared to the uninjured saline group (B). Total Lung Capacity (TLC‐30) is a sensitive indicator of lung injury. CASP‐administered mice showed reduced inflation volumes at 30 cm H_2_O inspiratory pressure than their controls group compliance (C). After CASP, mice were sacrificed at different time points, and ELISA determined BAL albumin concentrations (*n* = 9). Mice show increased lung injury following CASP administration compared to uninjured mice (D). Statistical significance of data was analyzed using one‐way ANOVA with Turkey's multiple comparison tests. (*p < 0.05 C57BL/6 uninjured vs. injured mice)

WT mice were subjected to CASP. The whole lung extracts (from injured and uninjured mice) were collected at different time points, and HIF‐1α expression was determined by western blot analysis. The levels of HIF‐1α expression in CASP‐challenged mice were higher than uninjured mice at all time points (Figure 1A).

Next, we examined the extent of mechanical injury. P–V curves were measured at various time points following acid and particle administration in C57BL/6 mice. The P–V curve data show that pulmonary compliance in the CASP‐administered mice was significantly less at 5, 24, and 48 h than the uninjured mice (Figure 1B). Total lung capacity (TLC‐30) is another parameter of lung compliance quantifying the volume that lungs can accommodate at 30 cm H_2_O of pressure. WT mice showed significantly reduced TLC at all times post CASP administration compared to uninjured mice (Figure 1C). Additionally, we examined BAL albumin levels using ELISA at 5, 24, and 48 h following CASP administration in WT mice. BAL albumin levels (an indicator of permeability injury) were significantly increased in CASP mice compared to the control mice (Figure 1D). These data suggest that CASP administration increased HIF‐1α expression and severe acute injury in WT mice.

### 3.2 ODD‐Luc mice show intense hypoxia, increased permeability injury, and inflammation following CASP administration

**FIGURE 2:**
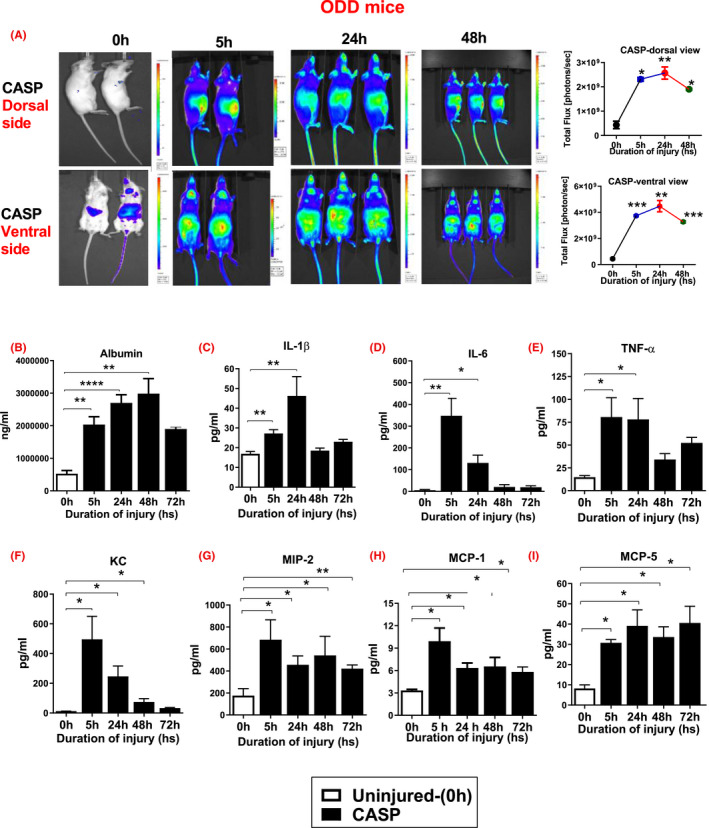
ODD‐Luc mice show hypoxia and increased injury and inflammation following CASP administration. ODD‐Luc mice and uninjured controls were subjected to IVIS imaging to measure the “degree of HIF‐1α protein stabilization after CASP.” ODD‐Luc mice showed increased luminescence at all time points after aspiration (A). After CASP, mice were sacrificed at different time points, and ELISA was used to determine the BAL albumin and cytokine concentrations. The levels of Albumin (B), IL‐1β (C), IL‐6 (D), TNF‐α (E), KC (F), MIP‐2 (G), MCP‐1 (CCL1) (H), and MCP‐5 (CCL12) (I), were measured following CASP. Higher injury and inflammation occurred following CASP administration in mice (*n* = 12). Statistical significance of data was analyzed using one‐way ANOVA with Turkey's multiple comparison tests. (*p <0.05 ODD‐Luc uninjured vs. injured mice)

The injury and inflammatory response resulting from CASP aspiration differ between mice of different genetic backgrounds.^53^, ^54^ We, therefore, investigated the inflammatory profile of *ODD*‐*Luc* mice, which are of an FVB background, following CASP‐induced lung injury to assess whether the injury is similar to that observed in C57BL/6 mice. For this, we examined the degree of hypoxia following CASP administration in mice. We used a mouse model in which a chimeric protein consisting of HIF‐1α oxygen‐dependent degradation domain fused to luciferase (ODD‐Luc) was ubiquitously expressed in all tissues. Naive and injured ODD‐Luc mice were then subjected to IVIS imaging (dorsal and ventral view) to measure the HIF‐1α stability at different time points following CASP (control and 5 h *n* = 2 per condition, 24 h and 48 h *n* = 3 per condition). Injured mice showed HIF‐1α at 5, 24, and 48 h (Figure 2A), which confirms that CASP administration causes hypoxic stress, leading to the accumulation of HIF‐1α in tissues. The bioluminescent signal is expressed and displayed as an intensity map. The image displayed is adjusted to provide optimal contrast and resolution without affecting quantitation based on the maximum photons per second that appeared at the 5 h time point. The statistical significance of data between the two groups was analyzed using a two‐tailed, unpaired *t*‐test with Welch's correction. Altogether, these data suggest that following CASP administration, hypoxic stress leads to the accumulation of HIF‐1α in all tissues.

Next, we investigated ODD‐Luc mice's injury and inflammatory profile following CASP. Bronchoalveolar lavage fluid was collected at different time points following CASP. ODD‐Luc mice BAL were analyzed by ELISA to measure the levels of permeability injury and pro‐ and anti‐inflammatory cytokines. The BAL albumin (a marker of permeability injury) was significantly higher at 5, 24, and 48 h time points compared to uninjured animals (0 h) (Figure 2B). The levels of interleukins (IL1‐β, IL‐6), tumor necrosis factor‐alpha (TNF‐α), monocyte chemotactic proteins (MCP‐5, MCP‐1), macrophage inflammatory protein (MIP)‐2, and KC were also measured. These cytokines reflect various attributes of acute inflammation. The levels of IL1‐β, IL‐6, and TNF‐α were all significantly elevated at 5 and 24 h following gastric aspiration (CASP) (Figure 2C–E). There were significant increases in keratinocyte chemoattractant (KC) levels at 5, 24, and 48 h following CASP (Figure 2F). The expression of MIP‐2 was significantly increased at 5, 24, 48, and 72 h after CASP (Figure 2G). Additionally, the levels of MCP‐1 and MCP‐5 were significantly increased at all relevant time points (Figure 2H,I).

### 3.3 ODD‐Luc mice show increased lung injury and pro‐inflammatory gene expression following CASP

**FIGURE 3:**
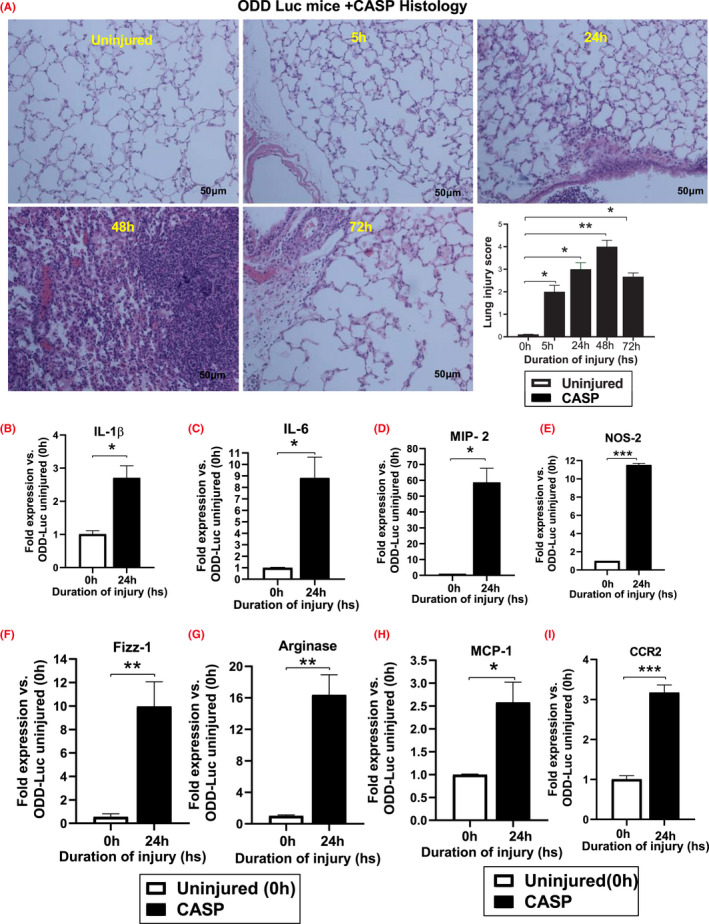
ODD‐Luc mice show increased injury and pro‐inflammatory gene expression following CASP. Histological staining of ODD mice lung sections (A). Histological figures confirmed increased lung injury in ODD‐Luc mice following CASP administration at all times compared with uninjured mice (*n* = 3). *Whole*‐*lung lysates* demonstrate significantly increased expression of inflammatory genes following acid aspiration. The expression of IL‐1β (B), IL‐6 (C), MIP‐2 (D), and NOS‐2 (E) Fizz‐1 (F), Arginase‐1 (G) MCP‐1 (H), and CCR2 (I) was significantly higher following acid‐exposed cells compared to uninjured cells. Data's statistical significance were analyzed using one‐way ANOVA with Turkey's multiple comparison tests (*p < .05 ODD‐Luc injured vs. uninjured ODD‐Luc mice)

Next, we examined hematoxylin and eosin‐stained lung tissue histopathological examination and measured the lung injury score. Histological evaluation revealed increased lung injury in ODD‐Luc mice at all times following CASP compared to uninjured mice (Figure 3A). Identical responses observed in terms of inflammation and injury data with non‐chimeric mice demonstrate that ODD‐Luc mice serve as a viable model to study CASP‐induced injury. Next, we examined the level of expression for pro‐inflammatory genes following CASP.

Whole RNA was isolated from the lung tissue, and the expression of IL1‐β, IL‐6, MIP‐2, NOS‐2 Fizz‐1, Arginase, MCP‐1, and CCR2 was measured following CASP administration using Q‐PCR. The expression of pro‐inflammatory genes such as IL1‐β and IL‐6 was significantly higher in the ODD‐Luc mice following CASP than uninjured mice at 24 h time points (Figure 3B,C). The levels of MIP‐2, NOS‐2, Fizz‐1, Arginase, MCP‐1, and CCR2 were significantly higher in the ODD‐Luc mice following CASP administration compared to control mice. (Figure 3D–I). Altogether, this data suggest that ODD‐Luc mice show greater injury and inflammation following CASP administration than the corresponding control mice.

### 3.4 The presence of HIF‐1α results in increased lung injury and inflammation following CASP

**FIGURE 4:**
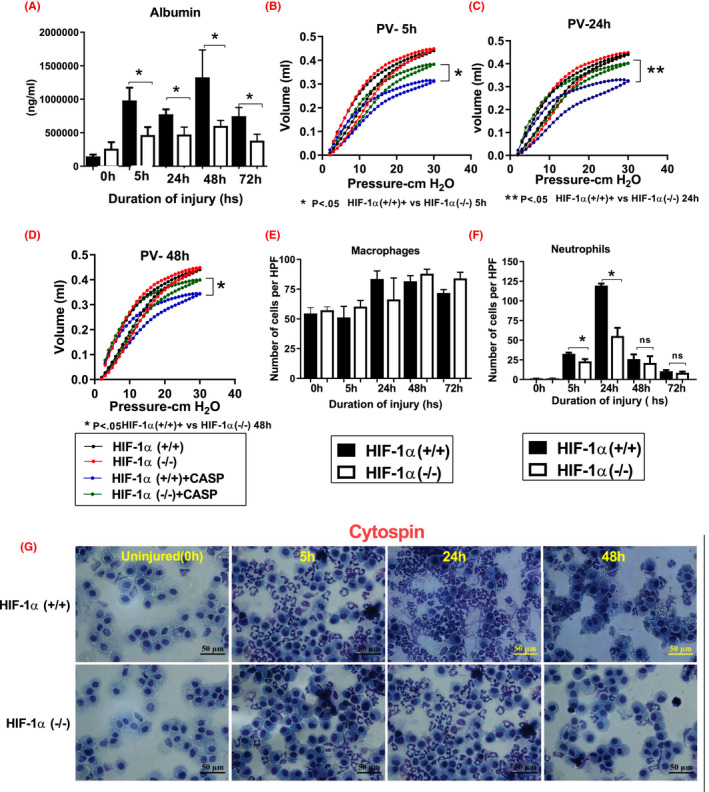
Injury and inflammation were reduced in HIF‐1α (−/−) mice following CASP. HIF‐1α (+/+) and HIF‐1α (−/−) mice (*n* = 12 per group) were subjected to CASP, and mice were sacrificed at 5, 24, 48, and 72 h time points. ELISA determined albumin concentration in the BAL. HIF‐1α (+/+) mice show a higher injury level following CASP (A). Closed‐chest pressure‐volume performance was measured at 5, 24, and 48 h after CASP (*n* = 6). Pulmonary compliance was higher in HIF‐1α (+/+) following CASP (B–D). There was no significant difference in the macrophages number in both groups following CASP injury (E), and neutrophils were significantly higher at 24 h in HIF‐1α (+/+) mice (F). Cytospins from BAL samples from HIF‐1α (+/+) and HIF‐1α (−/−) mice were stained with Giemsa reagent, and cell morphology was analyzed by light microscopy (*n* = 6 mice/group). Representative images of alveolar macrophages and neutrophils are shown (G). Statistical significance of data was analyzed using one‐way ANOVA with Turkey's multiple comparison tests (*p < 0.05 HIF‐1α (+/+) vs. HIF‐1α (−/−) mice)

We have determined that HIF‐1α plays a crucial role in mediating the acute inflammatory response following lung contusion.^14^, ^27^ To determine the functional aspects of HIF‐1α in the extent of injury and inflammation following CASP, we used HIF‐1α (+/+) and HIF‐1α (−/−) conditional knockout mice (*n* = 12). First, we examined BAL albumin level as an indicator of permeability injury. There was a significant reduction in the level of BAL albumin in HIF‐1α (−/−) mice at 5, 24, 48, and 72 h following acid and particle administration compared to HIF‐1α (+/+) mice (Figure 4A). Next, we examined the extent of mechanical injury. Pressure volume curves were measured at 5, 24, and 48 h time intervals following CASP. Pressure volume measurements indicated that pulmonary compliance was significantly decreased in the HIF‐1α (+/+) mice compared to the HIF‐1α (−/−) mice following CASP (Figure 4B–D). Next, we determined the levels of total macrophages and neutrophils in BAL fluid collected from both groups of mice at different time intervals using cytospin technique. The number of macrophages were not significantly different in 5, 24, 48, and 72 h time points in HIF‐1α (+/+) mice compared to HIF‐1α (−/−) mice (Figure 4E). The number of neutrophils were significantly higher at 24 h time points in HIF‐1α (+/+) mice compared to HIF‐1α (−/−) mice (Figure 4F,G). We have previously reported that neutrophils are mechanistically crucial in driving the acute inflammatory response following lung contusion.^29^, ^30^, ^55^, ^56^ Altogether, these data suggest that activation of HIF‐1α, specifically in type II AEC, contributes significantly to lung injury and acute inflammation following CASP.

### 3.5 HIF‐1α (−/−) mice show reduced inflammatory cytokines following CASP

**FIGURE 5:**
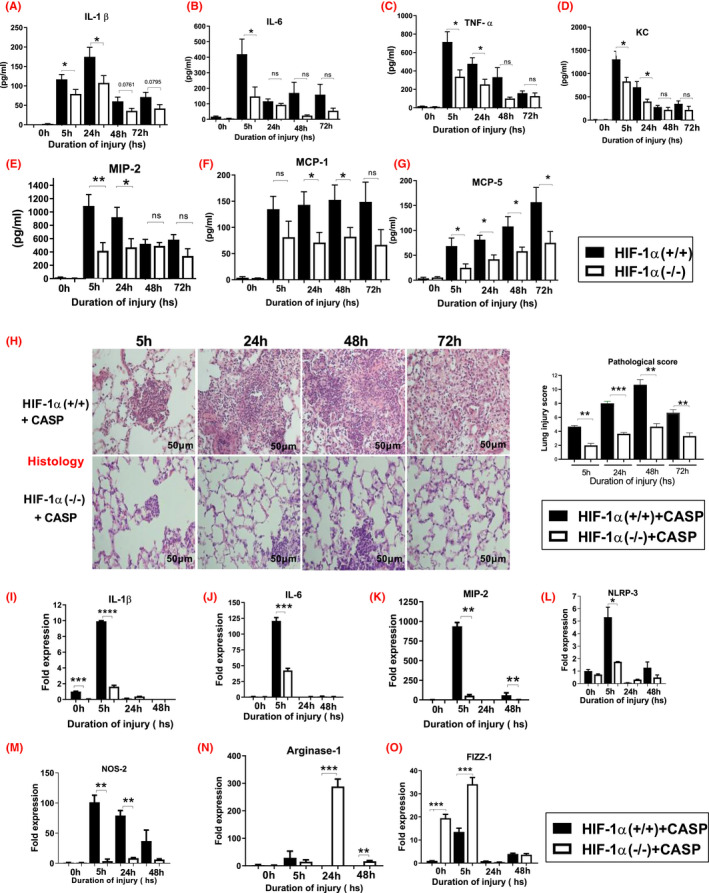
HIF‐1α (+/+) mice show increased injury inflammation compared to HIF‐1α (−/−) mice following CASP. HIF‐1α (+/+) and HIF‐1α (−/−) mice following aspiration mice were sacrificed at 5, 24, 48, and 72 h time points. The levels of various cytokines such as IL‐1β (A), IL‐6 (B), TNFα (C), KC (D), MIP‐2 (E), MCP‐1 (CCL2) (F), and MCP‐5 (CCL12) (G) were measured by ELISA. Overall, reduced inflammation was seen in HIF‐1α (−/−) mice following CASP administration (*n* = 12 per group). Representative histological features in HIF‐1α (+/+) and HIF‐1α (−/−) mice lung injured by deposition of CASP (*n* = 3 per group). The HIF‐1α (+/+) mice had significantly higher granuloma formation compared to HIF‐1α (−/−) mice (H, I). Statistical significance of data was analyzed using one‐way ANOVA with Turkey's multiple comparison tests (*p < 0.05 HIF‐1α (+/+) vs. HIF‐1α (−/−) mice). HIF‐1α (−/−) mice reduced pro‐inflammatory gene and increased M2 macrophages expression following CASP. RNA was isolated from the HIF‐1α (+/+), and HIF‐1α (−/−) mice whole lung extracts following CASP aspiration, and mice were sacrificed at 5, 24, and 48 h time points (*n* = 6 per group). Then, the levels of pro‐inflammatory mediator's interleukin (IL)‐1β (J), (IL)‐6 (K), MIP‐2 (L), and NLRP3 (M) were determined by real‐time PCR. Real‐time PCR results show a significant reduction in the pro‐inflammatory cytokine in HIF‐1α (−/−) mice. A quantitative reverse transcriptase‐polymerase chain reaction measured levels of NOS‐2 (M), Arginase‐1 (N), and FIZZ‐1 (O) genes. HIF‐1α (+/+) mice showed increased M1 (NOS‐2) macrophage polarization and reduced the M2 macrophages following aspiration (*n* = 6 per group). Statistical significance of data was analyzed using one‐way ANOVA with Turkey's multiple comparison tests (*p < 0.05 HIF‐1α (+/+) vs. HIF‐1α (−/−) mice). The fold expression of the 24 h acid and particles treated group compared the uninjured HIF‐1 α (+/+) mice (uninjured HIF‐1α (+/+) vs. 24 h post‐injury HIF‐1α (+/+))

Next, we examined the role of HIF‐1α activation on the production of pro‐/anti‐inflammatory mediators following CASP by using ELISA to measure the BAL levels of the interleukins (IL‐1β and IL‐6), tumor necrosis factor‐alpha (TNFα.), and chemokines such as keratinocyte chemoattractant (KC), macrophage inflammatory protein‐2 (MIP‐2), and monocyte chemotactic proteins (MCP‐1, MCP‐5). The levels of BAL IL‐1β were significantly higher at 5 and 24 h following CASP in the HIF‐1α (+/+) mice compared to HIF‐1α (−/−) mice (Figure 5A). The levels of IL‐6 in the BAL were significantly higher at 5 h following CASP in HIF‐1α (+/+) mice (Figure 5B). TNFα was also elevated at 5 and 24 h time points following CASP in the HIF‐1α (+/+) mice (Figure 5C). The levels of KC and MIP‐2 were also significantly elevated at 5 and 24 h following CASP in HIF‐1α (+/+) mice compared to HIF‐1α (−/−) mice (Figure 5D,E). The levels of MCP‐1 were significantly increased at 24 and 48 h following CASP in HIF‐1α (+/+) mice (Figure 5F). The levels of MCP‐5 were significantly increased at all the time points following CASP in HIF‐1α (−/−) mice compared to HIF‐1α (+/+) mice (Figure 5G). Histological figures confirmed increased lung injury in HIF‐1α (+/+) mice at all times compared with HIF‐1α (−/−) conditional knockout mice following CASP. An experienced, blinded pathologist evaluated mice from each group (*n* = 3) and examined for the presence of interstitial neutrophil infiltrate and intra‐alveolar hemorrhage (Figure 5H).

In an additional experiment, mice were subjected to CASP, RNA was isolated from the lungs of HIF‐1α (+/+) and HIF‐1α (−/−) mice (injured or uninjured animals). A quantitative reverse transcriptase‐polymerase chain reaction measured levels of IL‐1β, IL‐6, and MIP‐2 genes. These genes play a prominent role in the initiation of the acute inflammatory response following CASP. The expression of IL‐1β and IL‐6 was significantly reduced, at the 5 h time point, in the HIF‐1α (−/−) mice (Figure 5I,J). MIP‐2 expression was also significantly reduced at 5 and 48 h in the HIF‐1α (−/−) mice compared to HIF‐1α (+/+) mice after acid and particle aspiration (Figure 5K). Finally, expression levels of the NLRP3 inflammasome were found to be significantly decreased at 5 h in the HIF‐1α (−/−) compared to the HIF‐1α (+/+) mice (Figure 5L). These results suggest that the presence of HIF‐1α increases the inflammation resulting from acid particle administration.

### 3.6 HIF‐1α (−/−) mice show a reduction in pro‐inflammatory gene expression and better macrophage M2 phenotype polarization following CASP

Macrophage polarization into the M1 or M2 phenotype dictates an inflammatory response's nature, duration, and severity.^40^, ^41^, ^44^ Macrophage phenotypes can be characterized as pro‐inflammatory (M1) or immunomodulatory and tissue remodeling (M2). The expression of the M1 phenotype indicates a significant injury, whereas the M2 phenotype (also termed as alternatively activated) is associated with decreased production of pro‐inflammatory cytokines. Furthermore, high expression of the M2 phenotype is characterized by increased upregulation of the FIZZ‐1/Arginine pathway. HIF‐1α (+/+) and HIF‐1α (−/−) mice were subjected to CASP, and RNA was isolated from the lung. A quantitative reverse transcriptase‐polymerase chain reaction measured levels of NOS‐2, Arginase‐1, and FIZZ‐1 genes. The expression of NOS‐2 (pro‐inflammatory M1 macrophages) was elevated at all the time points in the HIF‐1α (+/+) mice compared to HIF‐1α (−/−) mice (Figure 5M).

On the other hand, the levels of (M2 macrophages phenotypes) Arginase‐1 (24 and 48 h) and FIZZ‐1 (0 and 5 h) were significantly higher in HIF‐1α (−/−) mice compared to HIF‐1α (+/+) mice (Figure 5N,O). Thus, we observed greater polarization toward the M2 phenotype in alveolar macrophages in HIF‐1α (−/−) mice and need further investigation. Another study from our labs shows that TLR3 deletion promotes a bias toward predominantly displaying M2 polarization in alveolar macrophages characterized by counter‐inflammatory gene products while maintaining a high phagocytic capability in both pneumonia and apoptotic cells compared to WT mice following pneumonia support this data.^44^

### 3.7 MCP‐1 neutralization attenuates granuloma formation in HIF‐1α (−/−) mice following CASP

**FIGURE 6:**
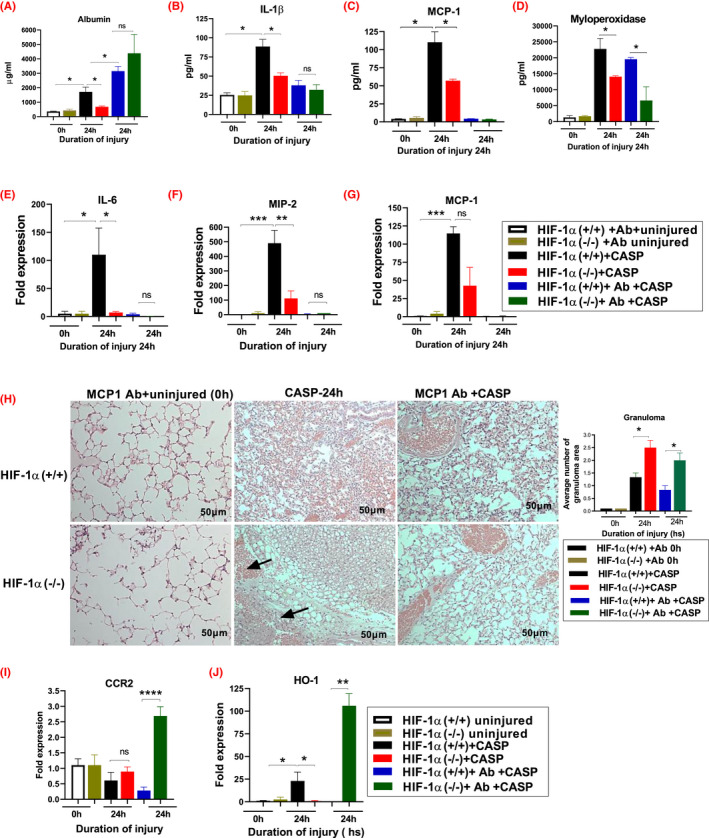
MCP‐1 neutralization abolishes the granuloma formation in HIF‐1α (+/+) mice following CASP. HIF‐1α (+/+) and HIF‐1α (−/−) mice were injected with MCP‐1 antibody 48 h before CASP, and mice were sacrificed at 24 h time points. Representative histology figures are shown for mice following CASP administration (*n* = 3) (H). A representative image of three similar images observed in four mice from each strain is shown. HIF‐1α (+/+) and HIF‐1α (−/−) mice following aspiration mice were sacrificed at 24 h time point. ELISA measured the levels of albumin (A) and various cytokines such as IL‐1β (B), MCP‐1 (CCL2) (C), and MPO (D). The gene expression of IL‐6 (E), MIP‐2 (F), MCP‐1 (CCL2) (G), CCR2 (I), and HO‐1 (J) was measured in HIF‐1α (+/+) and HIF‐1α (−/−) mice following aspiration. The fold expression of the 24 h acid and particles treated group compared the uninjured HIF‐1α (+/+) mice (uninjured HIF‐1α (+/+) vs. 24 h post‐injury HIF‐1α (+/+))

Previous reports show that after CASP aspiration, MCP‐1 knockout mice are associated with increased mortality and severely diffused pneumonia without evidence for granuloma formation in mice.^32^ In the first set of experiments, mice were subjected to the MCP‐1 antibody neutralization following CASP aspiration in the HIF‐1α (+/+) and HIF‐1α (−/−) mice. We examined BAL albumin levels using ELISA 24 h following CASP. BAL albumin levels were increased in MCP‐1 antibody neutralized HIF‐1α (+/+) and HIF‐1α (−/−) mice following CASP compared to corresponding controls. In contrast, the lung injury level was relatively higher in antibody‐administered HIF‐1α (−/−) and HIF‐1α (+/+) mice compared to corresponding control mice (Figure 6A). Next, we examined the role of pro‐inflammatory mediators following CASP using ELISA to measure the BAL interleukins (IL‐1β and IL‐6) and MCP1 and MPO. The levels of IL‐1β and MCP‐1 in the BAL were significantly higher at 24 h following CASP in the HIF‐1α (+/+) mice compared to HIF‐1α (−/−) mice. The IL‐1β and MCP‐1 in the BAL were significantly higher at 24 h following CASP in the HIF‐1α (+/+) mice compared to HIF‐1α (+/+) and HIF‐1α (−/−) uninjured mice. However, there was no significant difference in the MCP‐1 antibody neutralized HIF‐1α (+/+) mice compared to the HIF‐1α (−/−) mice following CASP administration (Figure 6B,C).

As expected, the BAL levels of myeloperoxidase expression were significantly higher at 24 h in the HIF‐1α (+/+) mice than HIF‐1α (−/−) mice. Conversely, the myeloperoxidase expression was higher in the antibody neutralized HIF‐1α (+/+) mice compared to HIF‐1α (−/−) mice (Figure 6D). The second set of experiments investigated the inflammatory gene expression in MCP‐1 antibody neutralized mice following CASP treatment. A quantitative reverse transcriptase‐polymerase chain reaction was performed to measure IL‐6, MIP‐2, MCP‐1, CCR2, and HO‐1 genes. The expression of IL‐6, MIP‐2, and MCP‐1 was significantly reduced at the 24 h time point in the HIF‐1α (−/−) mice compared to the HIF‐1α (+/+) mice. However, no gene expression could be detected in both mice after antibody neutralization following CASP treatment (Figure 6E–G).

Additionally, we performed histological evaluation using MCP‐1 antibody neutralization following CASP aspiration in both groups. The data shows that at 24 h post CASP aspiration, the HIF‐1α (+/+) mice had reduced granuloma formation compared to HIF‐1α (−/−) mice following CASP (Figure 6H). These results indicate that MCP‐1 plays an essential role in modulating inflammation in severe gastric aspiration pneumonia cases. Charo et al.^57^ reported that MCP‐1 mediates its effects through its receptor, CCR2, whose expression is relatively restricted to specific cell types. Consequently, we examined the role of CCR2 in granuloma formation in the presence and absence of the MCP‐1 antibody following CASP. We found significant CCR2 gene expression levels only in the MCP‐1 antibody‐administered HIF‐1α (−/−) mice compared to other tested groups (Figure 6I). These findings may reflect the differences in monocytes' chemotactic response versus macrophages and provide further evidence that the CCR2/MCP‐1 interaction is essential in these cells' functional regulation.

Heme oxygenase 1 (HO‐1) plays an essential role in anti‐inflammation, anti‐apoptosis, macrophage differentiation, and polarization. Previous studies show that HO‐1 promotes granuloma development and protects from pulmonary mycobacterial infections.^58^ Here, we examined the role of HO‐1 in regulating monocyte chemoattractant protein‐1 (MCP‐1) and chemokine receptor 2 (CCR2) in the presence or absence of the MCP‐1 antibody following CASP. A quantitative reverse transcriptase‐polymerase chain reaction measured levels of the HO‐1 gene. The expression of HO‐1 was elevated in the HIF‐1α (+/+) mice compared to HIF‐1α (−/−) controls. We found that the HO‐1 expression was significantly higher in HIF‐1α (−/−) mice that received MCP‐1 antibody following CASP aspiration compared to HIF‐1α (+/+) mice without MCP‐1 antibody (Figure 6J). These data suggest that the absence of MCP‐1 amplified the gene expression of CCR2 and HO‐1, diffused the granuloma formation, and increased the lung injury in the HIF‐1α (−/−) mice compared to HIF‐1α (+/+) mice.

### 3.8 NF‐kB activation is reduced HIF‐1α (−/−) mice following CASP

**FIGURE 7:**
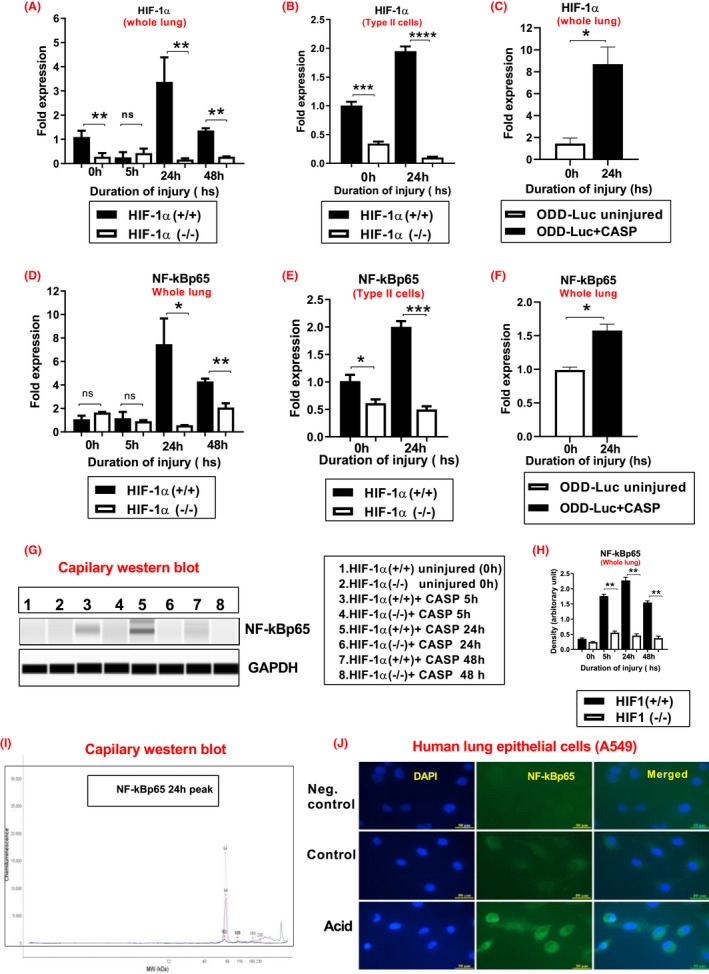
HIF‐1α (−/−) mice Type 2 AEC cells show a reduction in levels of HIF‐1 and NF‐kB after CASP. ODD‐Luc, HIF‐1α (+/+), and HIF‐1α (−/−) mice were subjected to CASP, and RNA was isolated from the uninjured and injured lungs (0, 5, 24, and 48 h), and type 2 alveolar epithelial cells (0 and 24 h). Then, the levels of HIF‐1α expression (A–C) and NF‐kBp65 expression (D–F) were determined by real‐time quantitative PCR (*n* = 6 per group). In a separate experiment, HIF‐1α (+/+) and HIF‐1α (−/−) mice were subjected to CASP, and lung extract was isolated at different time points. The expression of NF‐kBp65 was determined by capillary western blot (*n* = 3 per group). In HIF‐1α (+/+) mice, type 2 alveolar epithelial cells and whole lung extract increased NF‐kB levels following CASP (G–H). The capillary western blot detected the NF‐kBp65 signal with a molecular weight of 65 kDa (I). Following aspiration, the human lung epithelial cells showed significantly increased NF‐kBp65 expression compared to the controls (J). Statistical significance of data was analyzed using one‐way ANOVA with Turkeys multiple comparison tests (*p < 0.05 HIF‐1α (+/+) vs. HIF‐1α (−/−) mice and (*p < 0.05 ODD‐ Luc uninjured vs. injured mice). The fold expression of the 24 h acid and particles treated group compared the uninjured HIF‐1α (+/+) mice (uninjured HIF‐1α (+/+) vs. 24 h post‐injury HIF‐1α (+/+))

We have recently reported that HIF‐1α is a significant driver of acute inflammation after lung contusion and acid aspiration through type II AEC.^14^, ^27^, ^36^ In a separate experiment, mice ODD‐Luc HIF‐1α (+/+) and HIF‐1α (−/−) were subjected to acid and particle aspiration, and RNA was isolated from the lungs of injured and uninjured animals. A quantitative reverse transcriptase‐polymerase chain reaction measured levels of NF‐κB and HIF‐1α genes in the whole lung. The levels of HIF‐1α were significantly reduced in HIF‐1α (−/−) mice at 5, 24, and 48 h time points compared to HIF‐1α (+/+) mice (Figure 7A). This result also confirms that postnatal exposure to doxycycline can induce significant recombination of the HIF‐1α locus.

Additionally, to determine the activation on HIF‐1α in type 2 AEC in the injured lung, HIF‐1α (+/+) and HIF‐1α (−/−) mice were subjected to CASP, and the type II AEC were isolated (using anti‐CD32 and anti‐CD45 yield a pure AEC population) and measured at 0 and 24 h for HIF‐1α expression. The data confirm that the levels of HIF‐1α were significantly reduced in HIF‐1α (−/−) mice at 0 and 24 h time points compared to HIF‐1α (+/+) mice (Figure 7B). Moreover, the RNA isolated from the ODD‐Luc mice lungs and the HIF‐1α expression were analyzed by q‐PCR. The HIF‐1α expression was significantly higher in the ODD‐Luc mice at 24 h time points compared to uninjured mice (Figure 7C). The expression of NF‐κB (whole lung lysate) was found to be significantly elevated at 24 and 48 h time points in the HIF‐1α (+/+) mice following CASP compared to HIF‐1α (−/−) mice (Figure 7D). Additionally, to determine the precise regulation of NF‐κB activation on HIF‐1α in type 2 AEC in the injured lung, HIF‐1α (+/+) and HIF‐1α (−/−) mice were subjected to CASP, and the type II AEC were isolated and measured at 0 and 24 h for NF‐κBp65 expression. The data confirm that elevated expression of NF‐κB was seen in HIF‐1α (+/+) mice at 24 h following CASP compared to the HIF‐1α (−/−) mice (Figure 7E). Furthermore, the expression of NF‐κBp65 was significantly higher at 24 h in the ODD‐Luc mice following CASP than uninjured mice (Figure 7F). In a separate experiment, we examined NF‐κBp65 activation from whole lung lysates using western blot analysis. There was profound expression of NF‐κBp65 in HIF‐1α (+/+) mice at 5, 24, and 48 h following CASP compared to HIF‐1α (−/−) mice (Figure 7G,H). The NF‐κBp65 protein signal at 5 and 24 h with a molecular weight of 65 kDa was detected by capillary western blotting (Figure 7I). Next, we examined NF‐κBp65 activation in human lung epithelial cells. Cells were harvested 30 min following acid aspiration and subjected to NF‐κBp65 (green) and DAPI staining. The fluorescent images show intense NF‐κB staining in the lung epithelial cells following acid administration compared to the corresponding control (Figure 7J). This data suggest that HIF‐1α acts through a known secondary transcription factor, NF‐κB, suggesting crosstalk and interdependence between NF‐κB and HIF‐1α signaling cascades.

## 4 DISCUSSION

Pulmonary inflammation and progressive ARDS are commonly presented as acute shortness of breath with hypoxemia in clinical settings.^59^, ^60^ These gastric contents are variable and include secretions, small non‐acidified food particles (SNAP), and bile.^13^ Aspiration pneumonia is a pneumonitis resulting from foreign particles' entry (food particles) into the bronchial pathway.^61^, ^62^ ARDS is a severe condition characterized by fluid accumulation in the alveolar spaces from permeability resulting in atelectasis and hypoxemia.^59^, ^60^ Cases of acidic secretions and particulate aspiration (CASP) frequently lead to severe ARDS inflammation and permeability characteristics.^12^, ^13^ The severity of CASP injury ranges from mild, subclinical pneumonitis to progressive respiratory failure with high mortality and morbidity rates.^2^, ^4^, ^38^ Acid and particle‐induced injuries lead to an initial pro‐inflammatory environment that can resolve spontaneously in some patients. Inflammation is thus central to the pathogenesis and outcome of aspiration.^36^ This study aimed to define and characterize acute nonlethal lung injury pathogenesis after gastric, small particulate aspiration in the presence or absence of HIF‐1α expression.

Our lab has previously shown the importance of type II alveolar epithelial cells (AECs) and the critical role HIF‐1α plays in modulating the response to acid‐induced injury in these cells.^14^, ^36^ This study's importance was to expand these investigations of HIF‐1α to include the risk factor of gastric acid and food particulates. We first sought to confirm the injury and inflammatory profile that results from CASP injury. Following the CASP aspiration, C57BL/6 mice show increased damage, pulmonary compliance, and HIF‐1α expression (Figure 1). The data from ODD‐Luc mice show global hypoxia in all the explanted organs (liver, lung, kidney, and spleen [data not shown]), increased injury, and inflammation in direct association with CASP administration (Figure 2). Overall, our group's previous data demonstrate that gastric aspiration‐induced injury promotes HIF‐1α expression and activity. These data agree with our earlier findings that hypoxia is a prominent feature in other acute lung and acid aspiration‐induced injuries.^14^, ^36^ We recently reported on many potential activators of HIF‐1α and hypoxia markers, such as pimonidazole, which were confirmed via activation studies of HIF‐1α in lung injury models.^27^

By using HIF‐1α (+/+) and HIF‐1α (−/−) mice, our lab sought to elucidate the specific role of HIF‐1α in the inflammatory response to CASP. In the absence of HIF‐1α, markers of injury and inflammation were universally decreased in a significant manner. Albumin levels in bronchoalveolar lavage correspond to leakage and permeability of the lung tissue.^4^, ^32^, ^38^, ^39^, ^63^ In HIF‐1α (+/+) mice, albumin levels were increased significantly at all relevant time points, indicating a more severe permeability injury. The PV curve measurements and inflammatory cytokines were raised in the presence of HIF‐1α compared to HIF‐1α conditional knockouts (Figure 4). The data suggest that HIF‐1α has a significant role in driving heightened inflammation in response to CASP. Neutrophils act as “the ambulance” for the innate immune system and accumulate in large numbers immediately following injury or infection.^38^, ^64^, ^65^, ^66^ Their degranulation releases toxins and oxidants such as lactoferrin, myeloperoxidase, and gelatinase, which drive inflammation and injury. It has been shown that this harmful activity grows more severe in hypoxic conditions in lung tissue.^67^, ^68^ Moreover, HIF‐1α (+/+) mice were observed to contain increased numbers of neutrophil and macrophage infiltrates in alveolar spaces (Figure 4). MCP‐1 is a known recruiter of monocytic cells regulating granuloma formation in several subacute or chronic clinical pulmonary diseases.^69^ Our previous study found that MCP‐1 is essential for survival in pneumonitis and partly protects uninjured lung regions by promoting isolation and compartmentalizing tissue with active inflammation.^32^ Here, we found that MCP‐1 antibody neutralization reduces the HIF‐1α (−/−) mice's granuloma formation following aspiration than the HIF‐1α (+/+) mice. This data again confirms the importance of MCP‐1 in monocytic cell recruitment and granuloma formation in aspiration‐induced injury.

MCP‐1 mediates its effects via its receptor chemokine receptor 2 (CCR2), and unlike CCL2, CCR2 expression is relatively restricted to specific cell types. There are two alternatively spliced forms of CCR2 (CCR2A and CCR2B), which differ only in their C‐terminal tails.^70^ It is important to note that CCR2 plays a dual role, pro‐ and anti‐inflammatory functions. The pro‐inflammatory roles of CCR2 are dependent on APCs and T cells, while the anti‐inflammatory roles are dependent on CCR2 expression in regulatory T cells.^70^ Heme oxygenase‐1 (HO‐1) is an enzyme that protects cells from harmful reactive oxygen species and can control inflammatory responses. HO‐1 is induced by multiple stimuli such as oxidative stress, pro‐inflammatory cytokines, and upregulated lungs following mycobacterial infection.^58^ Monocyte chemotactic protein‐1 (MCP‐1/ CCL2), a C‐C chemokine, and its receptor, CCR2, on monocytes/macrophages are responsible for the recruitment of mononuclear cells from peripheral blood to sites of inflammation.^58^, ^71^ Here, we found that MCP‐1 neutralization increases CCR2 and HO‐1 expression in HIF‐1α conditional knockout mice compared to HIF‐1α (+/+) mice following CASP.

Macrophage infiltration is the other contributing factor affecting the severity of the CASP response. It is understood that macrophages can exhibit either an M1 phenotype characterized by the production of pro‐inflammatory cytokines and associated with a significant injury or an M2 (also termed alternatively active) phenotype associated with reparative and counter‐inflammatory tendencies. As might be expected, we observed an increase in the level of NOS‐2 expression (characteristic of M1 macrophages) and decreased levels of Arg‐1 and FIZZ‐1 expression (characteristics of M2 macrophages) in response to CASP injury (Figure 6). A potential role of HIF‐1α in response to injury inferred from the observed data includes the recruitment of primed immune cells and the maintenance of macrophage polarization toward a more inflammatory state.

As previously reported by our lab, HIF‐1α drives inflammation in LC through crosstalk with the secondary transcription factor NF‐κB.^14^, ^27^ The relationship between these two transcription factors is bidirectional through the phosphorylation of IκB and subsequent activation of NF‐κB, which has been shown to contribute to basal levels of HIF‐1α expression.^34^, ^35^ NF‐κB is a dimeric transcription factor that is a well‐characterized master regulator of hundreds of different genes, including pro‐inflammatory cytokines and chemokines.^72^, ^73^ However, we observed that when HIF‐1α was conditionally knocked out in type 2 AECs, the levels of transcript encoding NF‐κB dropped tremendously in both isolated type 2 AECs and in whole‐lung extracts (Figure 7). This data suggest a healthy interdependence between the two transcription factors in the context of CASP injury. Additionally, it highlights a mechanism by which HIF‐1α modulates the inflammatory and damage response to gastric aspiration via induction of pro‐inflammatory cytokines and chemokines secreted by the type 2 AECs. These cytokines, in turn, lead to the recruitment of activated neutrophils and M1 macrophages into the alveolar space.

In conclusion, the data presented in this manuscript demonstrates the significant role that HIF‐1α has in the innate immune response to CASP‐induced lung injury. Specifically, HIF‐1α (+/+) type 2 alveolar epithelial cells seem to clearly define the function in promoting the inflammatory response by establishing a bidirectional relationship between HIF‐1α and the transcription factor NF‐κB. The oxidants and noxious agents, such as nitric oxide, released by these immune cells, although helpful in fighting off secondary infections, could potentially contribute to the observed injurious effects associated with cases of ARDS where patients exhibit higher levels of inflammation, permeability injury, and complicated compliance symptoms. Consequently, blockading HIF‐1α would appear to be a promising alternative therapy to alleviate advanced ARDS cases, including those resulting from combined acid plus small particles (CASP) aspiration lung injury.

## CONFLICT OF INTEREST

None of the authors have a financial relationship with a commercial company.

## AUTHOR CONTRIBUTIONS

Conception and design: MVS and KR; Performed research: MVS, GY, TJ, SA, and AK; Analysis and interpretation: MVS, YS, and KR; Drafting the manuscript MVS, TJ, and KR’ for better clarity.
